# Anti-Cadherin-17 Antibody Modulates Beta-Catenin Signaling and Tumorigenicity of Hepatocellular Carcinoma

**DOI:** 10.1371/journal.pone.0072386

**Published:** 2013-09-11

**Authors:** Yonggang Wang, Felix H. Shek, Kwong F. Wong, Ling Xiao Liu, Xiao Qian Zhang, Yi Yuan, Ester Khin, Mei-yu Hu, Jian Hua Wang, Ronnie T. P. Poon, Wanjin Hong, Nikki P. Lee, John M. Luk

**Affiliations:** 1 Department of Oncology, Affiliated 6th People's Hospital, Shanghai Jiao Tong University, Shanghai, China; 2 Department of Surgery, The University of Hong Kong, Pokfulam, Hong Kong; 3 Department of Pharmacology and Department of Surgery, National University Health System, Singapore, Singapore; 4 Department of Radiology, Zhongshan Hospital, Fudan University, Shanghai, China; 5 Institute of Molecular and Cell Biology, A*STAR Singapore, Singapore; Seoul National University, Republic of Korea

## Abstract

Cadherin-17 (CDH17) is an oncofetal molecule associated with poor prognostic outcomes of hepatocellular carcinoma (HCC), for which the treatment options are very limited. The present study investigates the therapeutic potential of a monoclonal antibody (Lic5) that targets the CDH17 antigen in HCC. *In vitro* experiments showed Lic5 could markedly reduce CDH17 expression in a dose-dependent manner, suppress β-catenin signaling, and induce cleavages of apoptotic enzymes caspase-8 and -9 in HCC cells. Treatment of animals in subcutaneous HCC xenograft model similarly demonstrated significant tumor growth inhibition (TGI) using Lic5 antibody alone (5 mg/kg, i.p., t.i.w.; ca.60–65% TGI *vs*. vehicle at day 28), or in combination with conventional chemotherapy regimen (cisplatin 1 mg/kg; ca. 85–90% TGI). Strikingly, lung metastasis was markedly suppressed by Lic5 treatments. Immunohistochemical and western blot analyses of xenograft explants revealed inactivation of the Wnt pathway and suppression of Wnt signaling components in HCC tissues. Collectively, anti-CDH17 antibody promises as an effective biologic agent for treating malignant HCC.

## Introduction

Cadherins are first identified as cell adhesion molecules with roles in mediating cell-cell adhesion in biological processes, such as embryogenesis, development, organogenesis and differentiation. Recent studies have provided a strong link between tumorigenesis and cadherin deregulation [1]–[4]. Frequent loss of cadherin expression is observed in cancers especially for those cadherins with essential function in normal physiology. E-Cadherin, the first known cadherin molecule functioning in normal cell adhesion, experiences loss of expression in various types of cancers in liver [5], [6] and pancreas [7]. Despite that, other cadherins are known to involve in cancers differently. P-cadherin is another classical cadherin that has induced expression in breast cancers [8]. Similar to P-cadherin, N-cadherin is another notable one with overexpression in prostate cancers [9]. All these observations highlight the diversified roles of cadherins in tumorigenesis.

Cadherin-17 (CDH17) belongs to the non-classical cadherin with distinct structural feature of having seven cadherin repeats at its extracellular amino-terminus [10], [11]. Till now, Ksp-cadherin is another cadherin found sharing similar structure with CDH17 [12]. In addition to its distinct extracellular domain, CDH17 differs from classical cadherins in having a short cytoplasmic tail at its carboxyl-terminus [10]. No known interacting partner has been identified for CDH17 so far, except for a possible link with galectin-3 [11]. CDH17 was initially recognized as a peptide transporter [13] and recent evidences uncover its other function in cancers. The involvement of CDH17 in tumorigenesis is versatile depending on cellular context, such that either up-regulation or down-regulation of this molecule is found in different cancers, including pancreatic cancers, gastric cancers and colorectal cancers [11], [14]. Indeed, this deregulated expression of CDH17 in cancers suggests this molecule as clinical indicator for disease status and severity.

We and others have previously associated CDH17 with hepatocellular carcinoma (HCC) [15]–[20]. HCC is one of the most deadly cancers worldwide with an exceptional high rate in Asia and Africa [21]. Because of its aggressive nature, HCC patients usually have poor prognosis, leading to a similar number of incidence and death [21]. Surgery including partial hepatectomy and liver transplantation remains the frontline treatment for patients. Most patients are first diagnosed at late-stage and not amendable to tumor resection, while shortage of liver grafts limits the application of liver transplantation. Even for those patients after surgery, tumor recurrence is common and aggressive.

To search for new cures for aggressive HCC, we have observed an overexpression of CDH17 in HCC tumors and this abnormal expression is associated clinically to advanced tumor stage and presence of venous infiltration in these patients [15], [20]. RNA interference (RNAi) was then employed to show CDH17 possessing tumorigenic properties in HCC. We have also treated tumor-bearing nude mice with lentivirus harboring short hairpin RNA (shRNA) against CDH17 and this treatment resulted in regression of developing tumor xenografts [15]. All these data unequivocally demonstrate for the first time the clinical potential of targeting CDH17 as a treatment for HCC. Based on this proof-of-concept, we further develop monoclonal antibody (mAb) for potential application of CDH17-targeted therapy in HCC.

## Materials and Methods

### Human cell lines

Human HCC cell line MHCC97L and MHCC97H were gifts from Shanghai Fudan University, China [15]. The human gastric adenocarcinoma line IM95 was obtained from Japanese Collection of Research Bioresources (JCRB) cell bank. All cell lines were cultured as described [15], [22]. Cultured cells were incubated in Dulbecco's Modified Eagle Medium (DMEM) (Invitrogen, Carlsbad, CA) supplemented with 10% fetal bovine serum (FBS) (Invitrogen) and 1% penicillin-streptomycin (Invitrogen). Cultures were maintained in a humidified atmosphere at 37°C with 5% carbon dioxide.

### Production of mAb against CDH17 from mouse ascites

The immunization of BALB/c ByJ mice with recombinant extracellular domain 1–2 of human CDH17, fusion of antibody-producing cells with myeloma cells, and the subsequent selection of monoclonal hybridoma cells was performed as described [23], [24]. Clone designated as Lic5 was selected for experiments in this study. Approval for the animal study has been obtained from Committee on the Use of Live Animals in Teaching and Research from The University of Hong Kong.

To prepare Lic5 for various experiments, 8-week BALB/c nu/nu mice were injected with 500 µl paraffin oil for 7 days before they received an intraperitoneal injection of Lic5 hybridoma cells (5×10^5^ cells/mice in phosphate buffered saline, PBS). Ascites were collected 7–15 days after cell inoculation, which was followed by a second time collection 4–6 days after. Mouse ascites were centrifuged at 12,000× rpm at 4°C for 10 minutes and the supernatant was collected as monoclonal antibody. Antibody was then purified using Protein A Sepharose CL-4B (GE Healthcare, Piscataway, NJ) and desalted using Amicon Ultra-15 Centrifugal Filter Units (Millipore, Billerica, MA), following the protocols from manufacturers. Lastly, the concentration of the antibodies was estimated using RC DC Protein Assay (Life Science Research, Hercules, CA). Silver staining was then performed to show the purity of the antibody with procedure as described earlier [25]. The antibody immunoreactivity was confirmed using western blotting.

### *In vivo* efficacy study of Lic5

The anti-tumor effect of Lic5 was assessed using a murine subcutaneous tumor model developed as described [15]. In brief, 5-week BALB/c nu/nu mice were injected with 2×10^6^ MHCC97L cells in 200 µl PBS to form subcutaneous tumors in about 0.5 cm in diameter in 8 days. Tumor-bearing mice were then randomly divided into different treatment groups: *PBS*, sterile PBS; *IgG*, mouse IgG (5 mg/kg); *cisplatin*, 1 mg/kg cisplatin; *Lic5_L*, 2.5 mg/kg Lic5; *Lic5_H*, 5 mg/kg Lic5; and *Lic+cis*, a combined regimen of cisplatin (1 mg/kg) and Lic5 (5 mg/kg). Treatments were administered three times weekly for 4 consecutive weeks by intraperitoneal injection. Body weight and tumor size were estimated as before [15]. At the terminal time point, tumor xenografts derived by MHCC97L were resected for western blot and immunohistochemical staining to examine the protein levels of CDH17, proliferation marker Ki67, retinoblastoma (Rb) and Wnt/β-catenin pathway components. Lung tissues from all treatment groups were also harvested and studied for any metastatic tumors developed by means of staining with hematoxylin and eosin. Major organs like liver, kidney and spleen from Lic5-treated HCC tumor-bearing mice were isolated to examine for any signs of morphological damage after staining with hematoxylin and eosin.

### Western blotting

Procedure for western blot was performed as before [22], [26] using the following dilution of the primary antibodies: polyclonal goat anti-CDH17 (1∶000; Santa Cruz Biotechnology, Santa Cruz, CA), monoclonal rabbit anti-β-catenin (1∶1000; Cell Signaling Technology, Danvers, MA), polyclonal mouse anti-cleaved caspase-8 (Asp387) (1∶1000; Cell Signaling), polyclonal rabbit anti-cleaved caspase-9 (Asp353) (1∶1000; Cell Signaling), polyclonal rabbit anti-Rb (1∶200–1∶1000; Santa Cruz Biotechnology) and monoclonal rabbit anti-cyclin D1 (1∶200–1∶1000; Cell Signaling Technology).

### Immunohistochemistry

Immunohistochemistry was conducted as described earlier [27] using the following dilutions of primary antibodies: anti-CDH17 (1∶200), anti-β-catenin (1∶200), anti-cyclin D1 (1∶200), anti-Rb (1∶200) and anti-Ki67 (1∶500; Abcam, Cambridge, MA).

### Confocal microscopy

MHCC97H cells were seeded onto a glass-chambered slide (Millipore) at 20,000 cells/well. After treating cells with 100 µg/mL Lic5, cells were fixed with 3% paraformaldehyde before staining with either total or phospho-β-catenin (Thr41/Ser45) (Cell Signaling Technology) (1∶250) antibody for 1 hour. Bound antibodies were then detected using FITC or Alexa Fluor 488-tagged secondary antibody (1∶500). Stained cells were washed with PBS, treated with DAPI solution (0.1 µg/mL) and monitored under confocal microscope (Nikon, Melville, NY).

### Quantitative polymerase chain reaction (qPCR)

Total RNA extraction from cell lines received treatment and the subsequent first-strand cDNA synthesis were performed as described [25]. Gene expression assays were performed in duplicate using the Power SYBR Green PCR Master Mix (Applied Biosystems, Carlsbad, CA) in a 7900HT Fast Real-time PCR System (Applied Biosystems). Expression of the target was normalized by GAPDH expression.

### Statistical analysis

All statistical analyses on experimental data were performed using PRISM version 4.0 for Macintosh (GraphPad, San Diego, CA), with the significance between different groups calculated using ANOVA, Student's *t*-test, or chi-squared test in which a *p*-value<0.05 was considered as statistically significant.

## Results

### Lic5 targeting CDH17 reduced β-catenin level in human HCC cells

**Figure 1:**
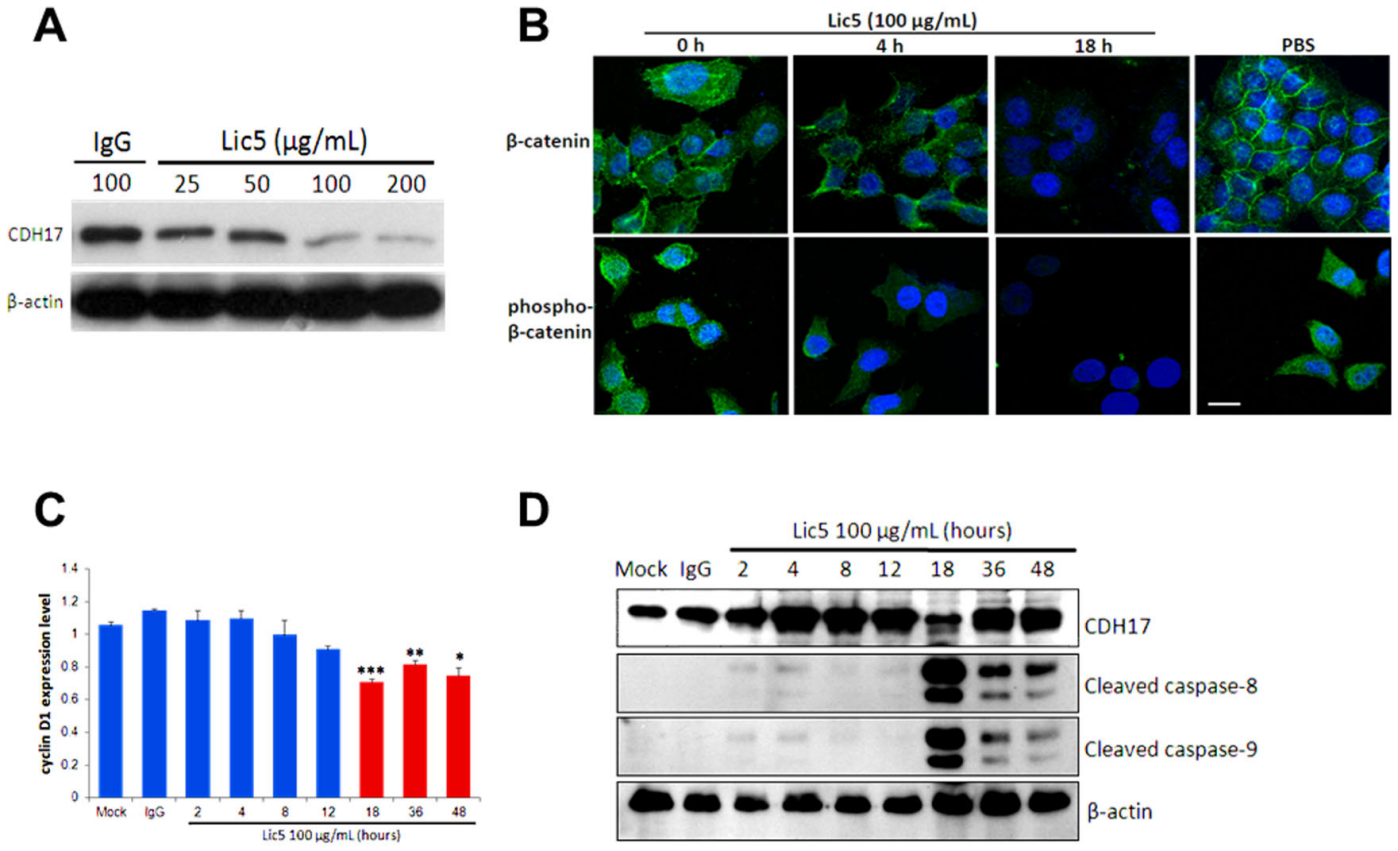
*In vitro* experiments of treating metastatic HCC cells with Lic5. Treatment of HCC cells with Lic5 inactivated CDH17/β-catenin signaling pathway and induced apoptosis. (A) MHCC97L cells with high level of CDH17 were treated with an increasing concentration of Lic5 from 25 to 200 µg/mL. A dose-dependent reduction in the protein level of CDH17 was detected using western blot. (B) Confocal microscopy images showed a reduction in cellular levels of total and phospho-β-catenin (at Thr41 and Ser45) proteins after Lic5 treatment in MHCC97H cells. PBS was used as a negative control. Scale bar, 20 µm. (C) Real-time qPCR showed a time-dependent reduction of cyclin D1 gene expression level in MHCC97H cells after treatment with Lic5 for 18, 36 and 48 hours.*, *p* = 0.001493; **, *p* = 0.00001424; ***, *p* = 0.00001377, when compared to the Mock (PBS) or IgG control. Shown are the representative set of data of three independent experiments, with each column represents the mean values of triplicate data. All *p*-values were calculated using ANOVA test of GraphPad PRISM. (D) Western blots showed treatment with Lic5 induced cleavage of caspase-8 and -9, but not by the Mock and IgG controls.

Our previous study showed that shRNA knockdown of CDH17 inactivated Wnt/β-catenin pathway in HCC [15]. To this end, we first examined whether Lic5, a mAb targeting the first two of the seven extracellular domains of CDH17 (Fig. S1) would attenuate the β-catenin signaling in metastatic human HCC cell line. We found Lic5 treatment caused substantial reduction of CDH17 protein in a dose-dependent manner (25 to 200 µg/mL) in MHCC97L cells (Fig. 1A). Confocal microscopy examination further revealed that treatment of MHCC97H cells with Lic5 for 18 hours completely suppressed the cellular levels of total β-catenin and those phosphorylated β-catenin at Thr41/Ser45, comparing to the no treatment control (Fig. 1B). Cyclin D1 is one of the transcriptional targets of β-catenin. We found that cyclin D1 mRNA level in Lic5-treated MHCC97H cells was significantly suppressed at 18, 36, and 48 hours after the onset of treatment (Fig. 1C). Notably, treatment of Lic5 was found to induce the activation cleavage of apoptosis-related molecules caspase-8 and caspase-9 at 18 hours and thereafter (Fig. 1D). Interestingly, a marked reduction of CDH17 protein level was observed at 18 hour (repeated experiments yielded similar results).

### Lic5 suppressed subcutaneous HCC tumor growth

**Figure 2:**
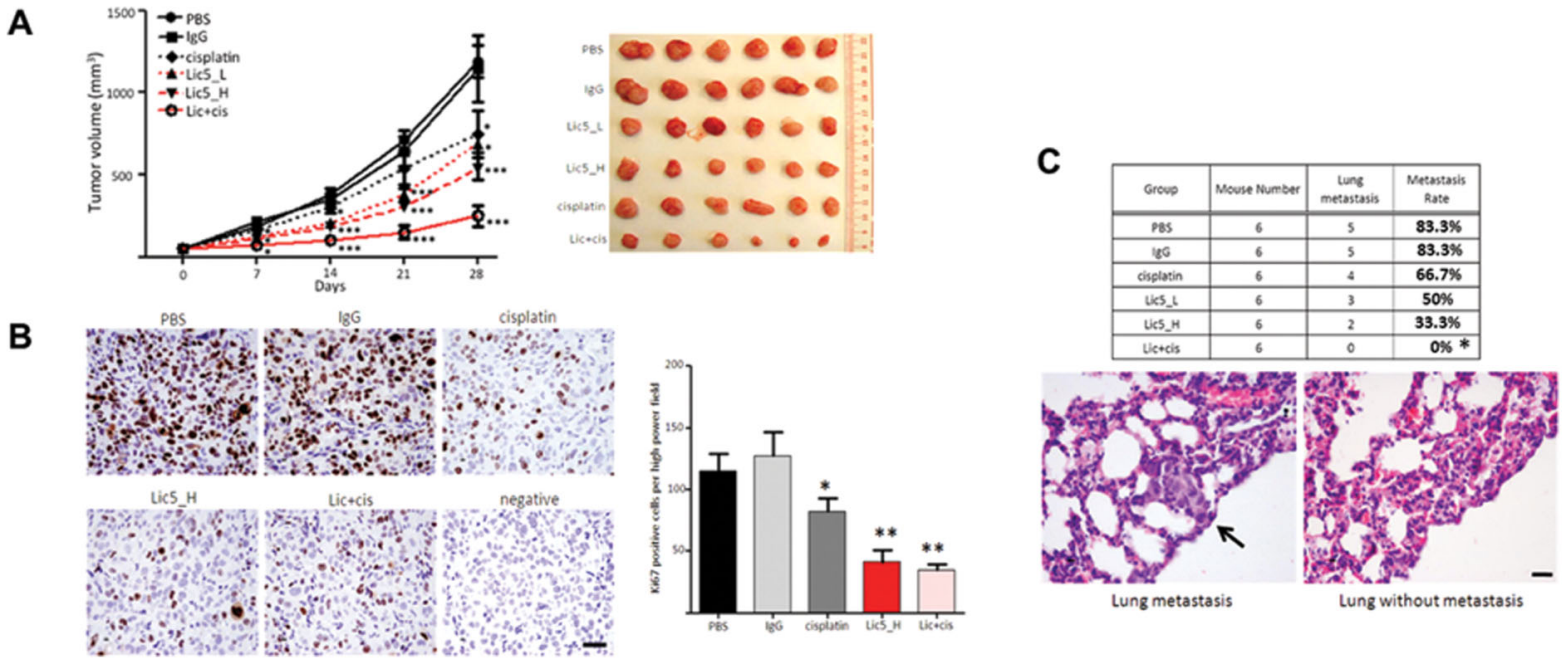
Antitumor and anti-metastatic properties of Lic5 in MHCC97L xenograft mouse model. Treatment of tumor-bearing nude mice with Lic5 inhibits tumor growth and sensitizes tumors to cisplatin. HCC subcutaneous tumors were developed in nude mice using CDH17-expressing MHCC97L cells. Tumor-bearing nude mice were then injected with Lic5 alone, 2.5 mg/kg (Lic5_L) or 5 mg/kg (Lic5_H), or in combination of 1 mg/kg cisplatin (Lic+cis).Mice of the control group received mouse IgG (5 mg/kg). All mice were injected three times weekly for four consecutive weeks. (A) Sizes of the subcutaneous tumors were estimated weekly throughout the experimental period (left panel) and subcutaneous tumors were resected 28 days after the onset of treatment (right panel). Reduction in the sizes of the tumors was observed in single treatment group (Lic5 or cisplatin). Combined regimen of Lic5 and cisplatin (Lic+cis) could result a complete inhibition on tumor growth. All *p*-values were calculated using Student's *t*-test of GraphPad PRISM, for which 6 tumor samples were included in each group for comparison between control and treatment groups. *, *p*<0.05 and ***, *p*<0.001. (B) The level of proliferating cells in HCC xenografts was studied using immunohistochemistry. A significant reduction in the number of Ki67-stained cells was observed in xenografts of mice injected Lic5, alone and in combination with cisplatin. Original magnification, ×400; scale bar, 80 µm. *, *p*<0.01 and **, *p*<0.001. (C) Lic5 treatment of tumor-bearing nude mice decreases the chance of developing metastatic tumors in lungs. Any tumors grew in lungs were spotted by staining with hematoxylin and eosin in series of sections derived from lung specimens. Treatment of these mice using Lic5 reduced the percentage of mice having metastatic tumors in lungs and the most significant effect was observed in combined treatment group with Lic5 and cisplatin. Chi-squared test was applied to compare control group with all other groups. *, *p*<0.05. Original magnification, ×400; scale bar, 40 µm.

We next examined whether Lic5 would suppress the growth of subcutaneous tumors derived by MHCC97L cells *in vivo*. Intraperitoneal injection of Lic5 could inhibit the growth of subcutaneous tumors, and at the terminal time point, Lic5 antibody at both high (5 mpk) and low (2.5 mpk) dosages was sufficient to yield >50% tumor growth inhibition (TGI) (Fig. 2A). Strikingly, combined regimen of Lic5 and cisplatin, a chemotherapeutic for cancer therapy, resulted in a nearly completed abrogation (>95% TGI) on HCC xenografts. The antibody treatment was believed to be safe because Lic5-treated mice did not suffer significant body weight loss (Fig. S2A). No sign of damages was observed in major/vital organs (i.e. liver, kidney, and spleen) of the Lic5-treated mice (Fig. S2B). In addition, the anti-tumor effect of Lic5 was similarly observed in a subcutaneous gastric cancer model that was derived from IM95 cells (Fig. S3).

HCC xenograft sections prepared from the PBS- or IgG-treated mice were heavily stained with the Ki67 proliferation marker (Fig. 2B, LHS). While cisplatin lowered the Ki67-positive cells by about 20% comparing to the PBS-treated mice, treatment with Lic5 antibody alone could achieve a reduction by 60%, comparable to the combined Lic+cis regimen (Fig. 2B, RHS). Collectively, these findings indicated Lic5 is an effective therapeutic agent to suppress HCC tumor growth in this HCC xenograft model, and its synergy with cisplatin or other clinically-used chemotherapeutics warrants further investigation.

Metastatic spread of cancer cells from the primary site to lungs is commonly seen in patients with HCC. Our animal model using MHCC97L recapitulated the clinical observations for 83.3% (5/6) of mice developed tumor metastasis in the lungs (Fig. 2C). Cisplatin treatment lowered the occurrence of developing tumor metastasis to 66.7% (*versus* 83.3% for controls). Lic5 treatment showed further reduction of lung metastasis (50% for 2.5 mpk *versus* 33.3% for 5 mpk). Most strikingly, combined Lic+cis treatment completely abrogated lung metastasis of MHCC97L (0 out of 6 animals)(Fig. 2C), exemplifying the anti-metastatic potential of Lic5 antibody by inhibiting the CDH17 functions in primary HCC tumor.

### Lic5 modulated Wnt/β-catenin pathway in subcutaneous HCC tumors

**Figure 3:**
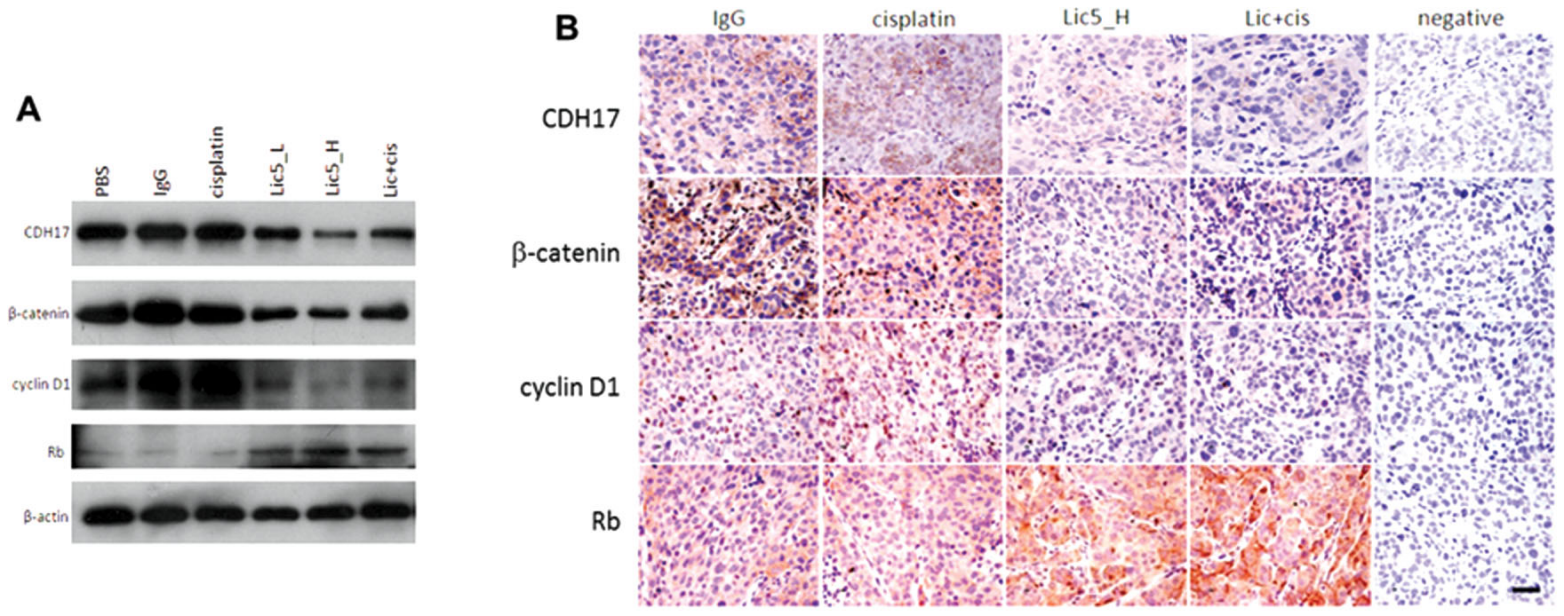
Lic5 treatment inactivated Wnt/β-catenin signaling in HCC tumors. Mice having MHCC97L-derived tumors were subjected to single or combined treatment of Lic5 and cisplatin as in Fig. 2. (A) Changes in the protein level of Wnt/β-catenin pathway components like β-catenin, cyclin D1 and retinoblastoma (Rb) in resected HCC tumors were detected using western blot. Suppression of CDH17 level using Lic5 accompanied with an inactivation of Wnt/β-catenin pathway, leading to reduction in the levels of β-catenin and cyclin D1 and induction in the level of Rb. β-Actin was used as a loading control. (B) Similar trend in the changes of the levels of β-catenin, cyclin D1 and Rb was observed in tissue sections of the HCC tumor xenografts using immunohistochemistry. Original magnification, ×400; scale bar, 80 µm.

We next investigated the cellular mechanism associated with the antitumor effect of Lic5 on subcutaneous HCC tumors. As shown by western blotting (Fig. 3A) and immunohistochemistry (Fig. 3B), Lic5 treatment reduced the protein levels of CDH17, β-catenin and its downstream cyclin D1 effector whereas expression of tumor suppressor Rb protein was induced. Of note, cisplatin treatment did not result in any major changes in the cellular level and localization of CDH17, β-catenin, cyclin D1 and Rb in tumor xenografts (Fig. 3A and 3B), for which cisplatin might go for different antitumor mechanism not directly impacting the Wnt pathway.

## Discussion

Recent studies have identified CDH17 as a promising therapeutic target for HCC treatment. In the present findings, we further demonstrated targeting CDH17 with a mAb Lic5 could specifically suppress the growth of CDH17-over-expressing subcutaneous tumors by inactivating Wnt/β-catenin pathway. Notably, the antibody treatment could markedly reduce metastatic spread of MHCC97L cells to the lungs. mAb against cancer-specific markers over-expressed on tumor cell surface promises an effective strategy of anti-cancer therapy [28], [29], for example, bevacizumab and trastuzumab for breast cancer [30], [31] and cetuximab for colon, and head and neck cancers [32], [33]. In particular, trastuzumab or Herceptin that targets the human epidermal growth factor receptor 2 (HER2)-positive breast tumors specifically has been successfully applied in the clinics to benefit a subgroup of cancer patients of HER2-positive cancer [31]. Thus far effective biologics agents for clinical treatment of HCC have not been available. Our finding suggests Lic5 would be a promising candidate that can be further developed into a new biological agent for potential therapy of advanced HCC.

Our previous studies have demonstrated CDH17 as an oncofetal molecule with confirmed tumor-inducing potentials and tumorigenic properties in HCC that Wnt/β-catenin pathway is its downstream pathway leading to tumorigenesis [15], [34]. This study using mAb against CDH17 provided supporting evidence showing CDH17 as an upstream molecule of Wnt/β-catenin pathway. Here we further demonstrated that using anti-CDH17 mAb showed antitumor effects alone and in combination with cisplatin chemotherapy regimen. Lic5 recognizes the extracellular domain 1–2 of CDH17. The functional role of these domains has remained unclear, however for their high resemblance with respective structural domains of classical cadherins [35], domain 1–2 of CDH17 is believed to function in cell adhesion similar to those of classical cadherins. Whether Lic5 would affect the cell adhesive function of cancer cells remains to be further characterized, but our findings clearly suggested targeting the extracellular domain of CDH17 by Lic5 can inactivate the Wnt/β-catenin pathway. The action of Lic5 shows some degree of similarity to other therapeutic antibodies. It has been reported that a mAb targeting extracellular domain of N-cadherin can trigger anti-tumorigenic response in prostate cancer cells [36]. In addition, like ramucirumab against vascular endothelial growth factor receptor 2 (VEGFR2) [37] and trastuzumab against HER2 [38], Lic5 demonstrates its anti-cancer effect by acting on the upstream component of signaling pathway essential to tumor progression. Some other drugs target deregulated pathways or processes directly and examples include those against proteasome for protein degradation [39] and Hedgehog pathway [40]. For HCC, a plethora of molecular-based drugs belonging to different categories as above was under clinical trial phase for testing to be used in clinics [41].

No matter how good the efficacy of a given anticancer agent, other aspects like side-effects and delivery methods should be weighted carefully before a drug can be used as a medicine for treating patients. Importantly, the chance of tumors to develop drug resistance should be taken into account for consideration of long-term drug treatment. In this study, treatment of Lic5 seems to be specific to CDH17-expressing HCC cells and xenografts only as no significant changes in the phenotype of those cells with low level of CDH17 and morphology of other major organs were observed after the treatment period. Besides, the body weight loss in animals subjected to this treatment is minimal, excluding the possibilities of severe side-effects occurred. The next attempt for this study is to synthesize humanized forms of these antibodies for further proof of the concepts and confirmation of the safety profiles [42], [43].

## Supporting Information

Figure S1 Characterization of Lic5. (A) The high purity of Lic5 was revealed by silver staining showing two stained bands corresponding to the light and heavy chains of the antibody. (B) Western blot using Lic5 was performed. A 120-kDa band corresponding to immuno-reactive CDH17 was detected in CDH17-expressing MHCC97L cells. (C) Time-kinetic confocal microscopy of Lic5 antibody localization against OCUM-1 cell line. FITC-conjugated Lic5 mAb was allowed to incubate with OCUM-1 cells at 5, 10 and 30 min in Ab/serum free DMEM medium (1∶500), washed thrice, and fixed. The green fluorescent stain revealed strong intracellular signal of Lic5 at 30 min after incubation, compared to the peripheral nature of the staining at time 5 min.
(TIF)

Figure S2 Evaluation of Lic5 safety in nude mice. (A) Treatment of tumor-bearing nude mice with Lic5 did not associate with loss of body weight, while cisplatin treatment hampered the body weight of mice. Combined treatment of Lic5 and cisplatin rescued the weight loss caused by cisplatin. (B)Treatment of HCC tumor-bearing nude mice with Lic5 does not associate with tissue damage of major organs. Hematoxylin and eosin staining was performed in tissue sections prepared from liver, kidney and spleen isolated from mice treated with Lic5. No morphological damage was found in these organs. Original magnification, ×100 (upper panel), ×200 (lower panel); scale bar, 120 µm.
(TIF)

Figure S3 Effect of Lic5 on *in vivo* IM95 gastric cancer model. Gastric cancer subcutaneous tumors were developed in nude mice using CDH17-expressing IM95 cells. Tumor-bearing nude mice were injected with Lic5 alone (Lic5_H, 5 mg/kg), or in combination of 1 mg/kg cisplatin (Lic+cis). Mice of the control group received mouse IgG (5 mg/kg). All mice were injected three times weekly for four consecutive weeks. (A) Sizes of the subcutaneous tumors were estimated weekly throughout the experimental period (left panel) and subcutaneous tumors were resected 28 days after the onset of treatment (right panel). Reduction in the sizes of the tumors was observed in single treatment group (Lic5 or cisplatin). Combined regimen of Lic5 and cisplatin (Lic+cis) could result a complete inhibition on tumor growth. (B) Treatment of tumor-bearing nude mice with Lic5 did not associate with loss of body weight, while cisplatin treatment hampered the body weight of mice. Combined treatment of Lic5 and cisplatin rescued partially the weight loss caused by cisplatin.
(TIF)
