# Supplementary figures and images for: Anti-Cadherin-17 Antibody Modulates Beta-Catenin Signaling and Tumorigenicity of Hepatocellular Carcinoma
Source: PLoS One. 2013 Sep 11;8(9):e72386. doi: 10.1371/journal.pone.0072386 (PMC3770615; doi:10.1371/journal.pone.0072386)

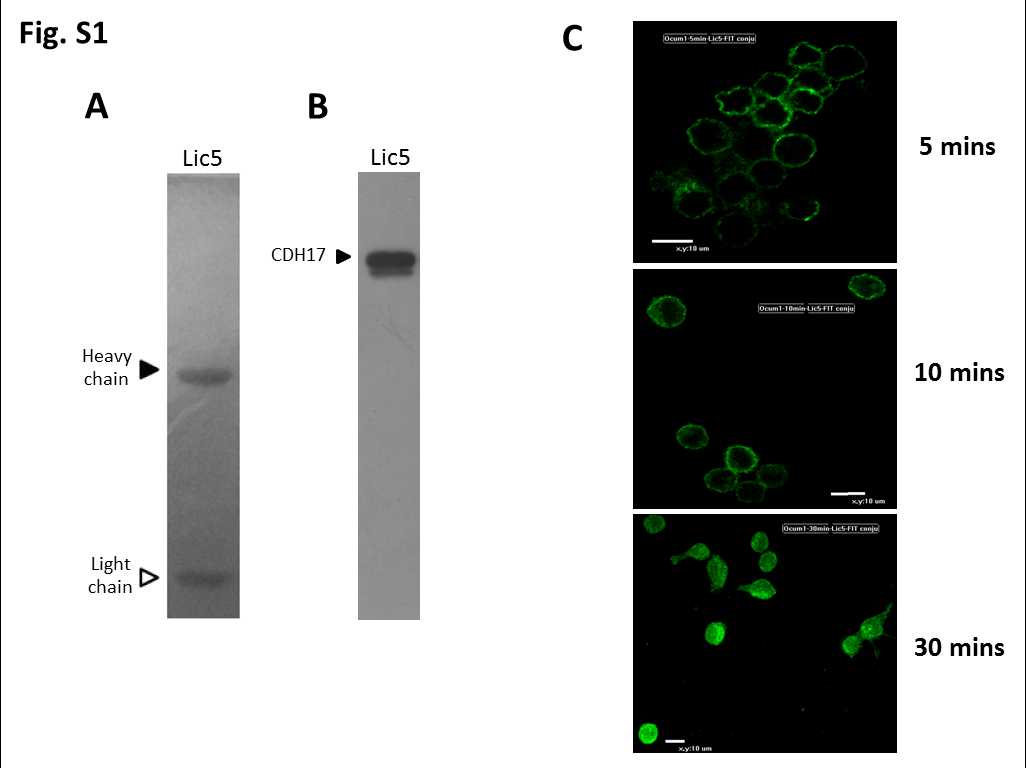

Supplement: Figure S1 — Characterization of Lic5. (A) The high purity of Lic5 was revealed by silver staining showing two stained bands corresponding to the light and heavy chains of the antibody. (B) Western blot using Lic5 was performed. A 120-kDa band corresponding to immuno-reactive CDH17 was detected in CDH17-expressing MHCC97L cells. (C) Time-kinetic confocal microscopy of Lic5 antibody localization against OCUM-1 cell line. FITC-conjugated Lic5 mAb was allowed to incubate with OCUM-1 cells at 5, 10 and 30 min in Ab/serum free DMEM medium (1∶500), washed thrice, and fixed. The green fluorescent stain revealed strong intracellular signal of Lic5 at 30 min after incubation, compared to the peripheral nature of the staining at time 5 min. (TIF) [file pone.0072386.s001.tif]

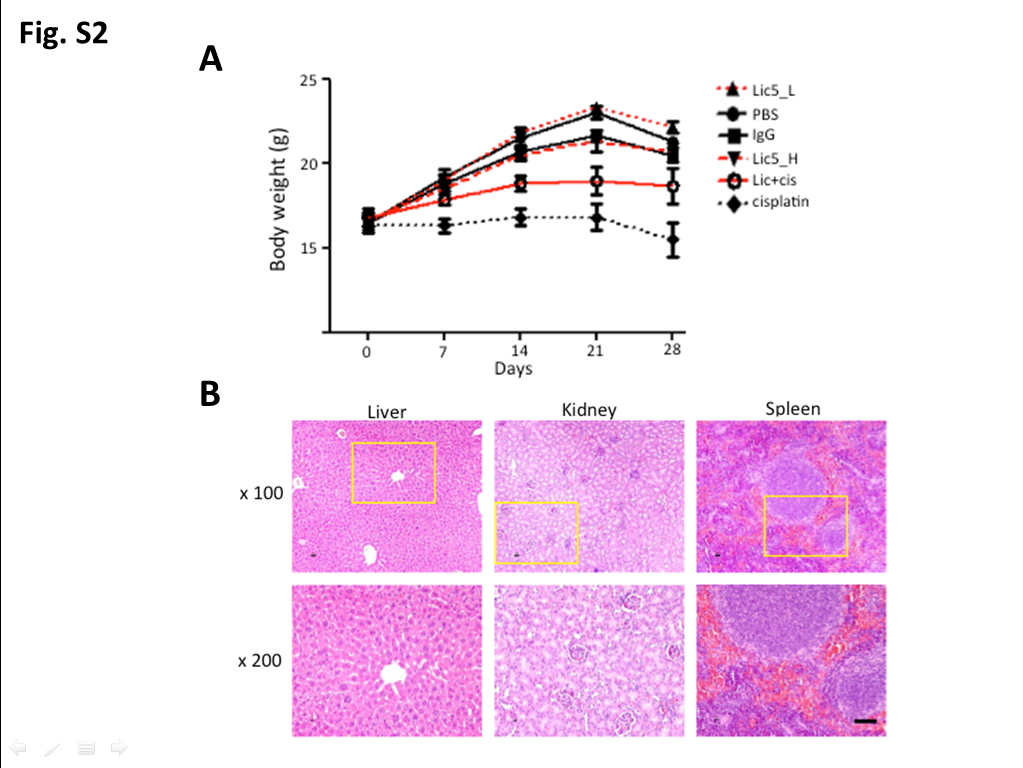

Supplement: Figure S2 — Evaluation of Lic5 safety in nude mice. (A) Treatment of tumor-bearing nude mice with Lic5 did not associate with loss of body weight, while cisplatin treatment hampered the body weight of mice. Combined treatment of Lic5 and cisplatin rescued the weight loss caused by cisplatin. (B)Treatment of HCC tumor-bearing nude mice with Lic5 does not associate with tissue damage of major organs. Hematoxylin and eosin staining was performed in tissue sections prepared from liver, kidney and spleen isolated from mice treated with Lic5. No morphological damage was found in these organs. Original magnification, ×100 (upper panel), ×200 (lower panel); scale bar, 120 µm. (TIF) [file pone.0072386.s002.tif]

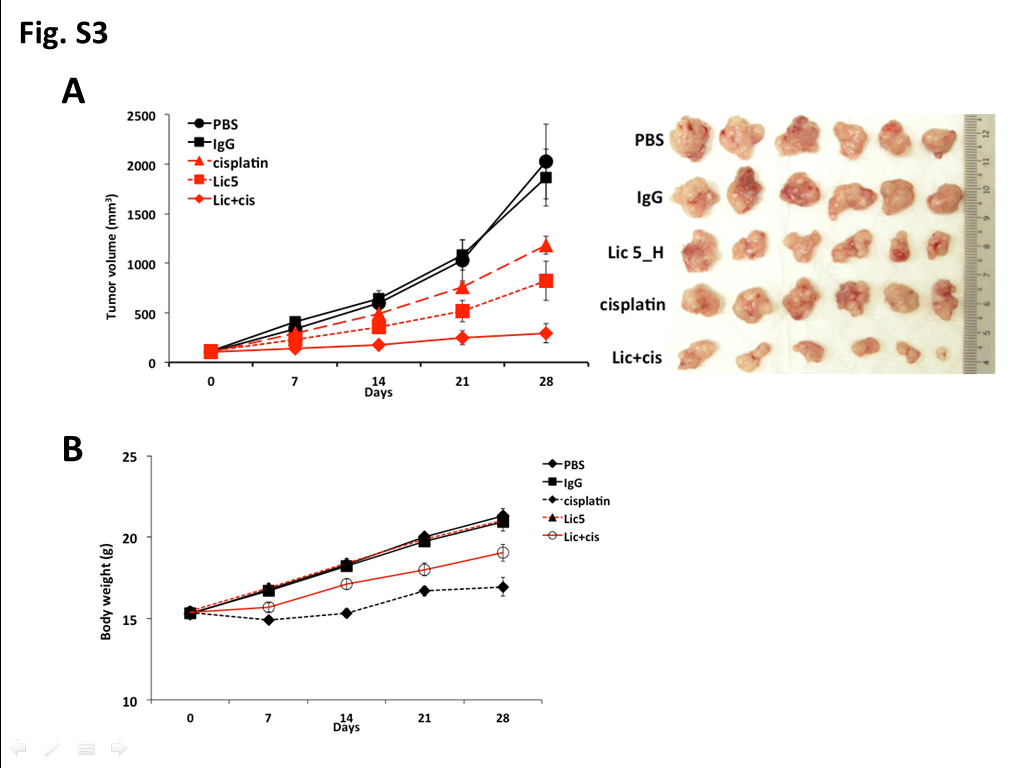

Supplement: Figure S3 — Effect of Lic5 on in vivo IM95 gastric cancer model. Gastric cancer subcutaneous tumors were developed in nude mice using CDH17-expressing IM95 cells. Tumor-bearing nude mice were injected with Lic5 alone (Lic5_H, 5 mg/kg), or in combination of 1 mg/kg cisplatin (Lic+cis). Mice of the control group received mouse IgG (5 mg/kg). All mice were injected three times weekly for four consecutive weeks. (A) Sizes of the subcutaneous tumors were estimated weekly throughout the experimental period (left panel) and subcutaneous tumors were resected 28 days after the onset of treatment (right panel). Reduction in the sizes of the tumors was observed in single treatment group (Lic5 or cisplatin). Combined regimen of Lic5 and cisplatin (Lic+cis) could result a complete inhibition on tumor growth. (B) Treatment of tumor-bearing nude mice with Lic5 did not associate with loss of body weight, while cisplatin treatment hampered the body weight of mice. Combined treatment of Lic5 and cisplatin rescued partially the weight loss caused by cisplatin. (TIF) [file pone.0072386.s003.tif]
